# Subcutaneous abscess caused by *Streptococcus pneumoniae* serotype 28F in an infant: a case report

**DOI:** 10.1186/s12887-020-02465-3

**Published:** 2021-01-04

**Authors:** Tomohiro Hirade, Ai Harada, Daisuke Koike, Yasuhiro Abe, Tsuyoshi Higuchi, Fumihide Kato, Bin Chang, Akiyoshi Nariai

**Affiliations:** 1 Department of Pediatrics, Shimane Prefectural Central Hospital, 4-1-1 Himebara, Izumo, 693-8555 Shimane Japan; 2 Department of Bacteriology I, National Institute of Infectious Diseases, Toyama 1-23-1, Shinjuku-ku, 162-8640 Tokyo, Japan

**Keywords:** Invasive pneumococcal disease, Non-vaccine serotype, Subcutaneous abscess

## Abstract

**Background:**

Invasive pneumococcal disease (IPD) is defined by the detection of *Streptococcus pneumoniae* on culture from samples obtained from a normally sterile site. Pneumococcal conjugate vaccines (PCV) have been developed for the prevention of IPD that is caused by highly virulent serotypes. Despite the effective reduction of IPD caused by vaccine serotypes after the introduction of PCV, there has been a rapid increase in the incidence of IPD caused by non-vaccine serotypes, and serotype replacement has become a global issue.

**Case presentation:**

We report a previously healthy 4-month-old girl presenting with a large subcutaneous abscess caused by *S. pneumoniae*, identified as non-vaccine serotype 28F. The patient had received routine vaccination, including PCV vaccination. After the incision and drainage of the subcutaneous abscess, the patient was treated with antibiotics. She was discharged on Day 7 of hospitalization without any residual sequelae.

**Conclusions:**

Subcutaneous abscess is a common pediatric skin and soft tissue infection, whereas pneumococcal subcutaneous abscesses are quite rare. As the pneumococcal serotype 28F caused a subcutaneous abscess, this serotype possibly has a high virulence. The incidence of IPD caused by non-vaccine serotypes, such as 28F, is expected to increase in the future. The consolidation of international data on pneumococcal serotypes is important for the development of novel PCV.

## Background

There are more than 90 known serotypes of *Streptococcus pneumoniae*, and each produces a unique polysaccharide capsule that protects the bacterium against the host immune effectors [1, 2]. Serotypes that are more heavily encapsulated tend to have higher carriage prevalence and greater virulence [3].

Invasive pneumococcal disease (IPD) is defined by the detection of *S. pneumoniae* on culture from samples obtained from a normally sterile site [4]. Pneumococcal conjugate vaccines (PCV) have been developed for the prevention of IPD that is caused by highly virulent serotypes. Despite the effective reduction of IPD caused by vaccine serotypes after the introduction of PCV [10- or 13-valent PCV (PCV13)], there has been a rapid increase in the incidence of IPD caused by non-vaccine serotypes [1, 4–10].

We report a previously healthy 4-month-old girl presenting with a subcutaneous abscess on the left thigh caused by *S. pneumoniae.* The pneumococcal serotype was identified to be the non-vaccine serotype 28F. This is the first case report of IPD due to non-vaccine serotype 28F.

## Case presentation

**Fig. 1:**
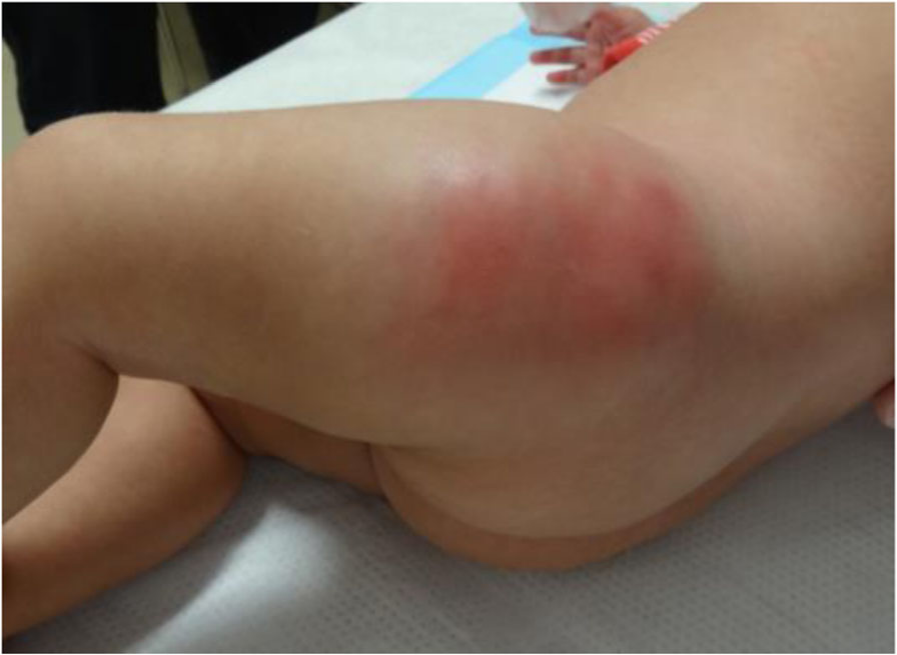
Erythematous skin lesion at hospitalization

**Fig. 2:**
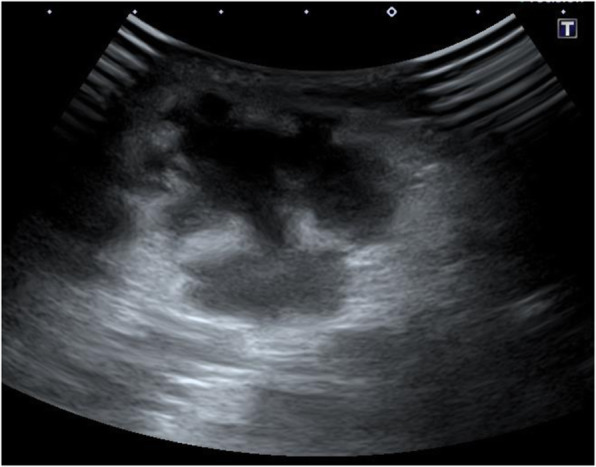
Ultrasonographic imaging: Ultrasonographic image of the affected skin shows a hypoechoic lesion

**Fig. 3:**
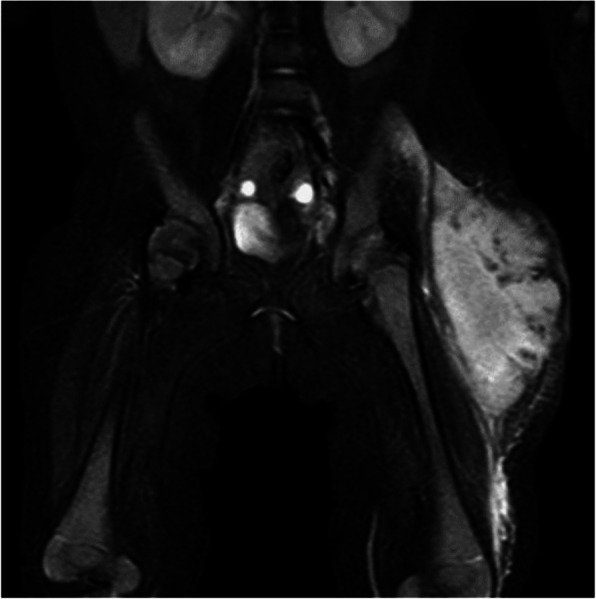
Magnetic resonance imaging: Fat-suppressed T2-weighted image shows a large subcutaneous fluid collection in the left thigh

A 4-month-old girl was hospitalized with a swelling on her left thigh and an inability to move her left leg; she was afebrile. The patient was born by normal vaginal delivery after a full-term gestation and had no relevant medical history. The swelling of the left thigh appeared 1 week before hospitalization and gradually increased in size. The patient had received routine vaccination, including a second PCV-13 vaccination on her left thigh 1 month prior to hospitalization. After the vaccination, the patient did not have fever, and the injection site was clear of any signs of infection; therefore, she was not followed up. On examination, the patient appeared alert and well. She had normal vital signs (temperature, 36.1 °C; pulse, 152 beats per min; respiratory rate, 30 breaths per min; and oxygen saturation, 99%). The patient did not move her left leg and cried on touching it. An erythematous, tender, and firm, but not fluctuant, skin lesion measuring 8 × 7 cm was detected on her left thigh (Fig. 1). Laboratory investigations revealed leukocytosis (white blood cell count, 24.2 × 10^9^ /L; 47% neutrophils, 42% lymphocytes); hemoglobin concentration, 11.6 g/dL; platelet count, 910 × 10^9^ /L; and C-reactive protein level, 35.9 mg/L. Immunoglobulin (Ig) levels were as follows: IgG, 2,274 mg/dL; IgA, 99 mg/dL; and IgM, 99 mg/dL. The IgG subclasses, complement studies as well as neutrophil sterilizing and phagocytosis functions were normal. HIV screening showed negative results. Ultrasonographic imaging revealed a large subcutaneous hypoechoic fluctuant fluid-filled lesion measuring 8 × 7 × 4 cm (Fig. 2). There were no hypoechoic lesions in the abdominal organs. Magnetic resonance imaging revealed a large collection of subcutaneous fluid on the left thigh, with absence of other diseases such as osteomyelitis or pyomyositis (Fig. 3).

Based on her clinical symptoms and the imaging findings, the patient was diagnosed with a subcutaneous abscess. Incision of the subcutaneous abscess resulted in the drainage of a large volume of purulent material. Gram staining of the pus showed encapsulated gram-positive diplococci. The patient was treated with intravenous panipenem/betamipron (PAPM/BP). *S. pneumoniae* was isolated from the pus, while cultures from blood, skin, and nasopharyngeal samples were all negative. Because the pneumococcal isolate was susceptible to penicillin, PAPM/BP was replaced by intravenous ampicillin on Day 4 of hospitalization. The patient was discharged on Day 7 of hospitalization after full resolution of her illness without any residual sequelae.

The pneumococcal isolate was sent to the National Institute of Infectious Diseases (Tokyo, Japan) and was identified as serotype 28F based on observation of Quellung capsule swelling. The serotype 28F was susceptible to penicillin, cephalosporins, carbapenems, quinolones, and macrolides.

## Discussion and conclusions

The introduction of PCV has been followed by rapid increase in IPD caused by non-vaccine serotypes, and serotype replacement has become a global issue. Serotype replacement varies slightly across countries or regions [1, 4–10]. In Japan, the seven-valent PCV (PCV7) (in children < 5 years) was introduced in February 2010 and was replaced by PCV13 in November 2013. The recommended PCV13 schedule indicates vaccination at 2, 4, 6, and 12–15 months of age. The PCV13 vaccination rate throughout Japan has been estimated at > 95% following the introduction of routine PCV-13 vaccination [9]. In a Japanese nationwide surveillance of pneumococcal infections in pediatric patients undertaken from 2010 to 2017, the most frequently identified causative serotypes of IPD were 24F, 15A, and 12F [9]. In the UK, PCV7 was introduced in September 2006 and was replaced by PCV13 in April 2010. From 2000 to 2017, IPD due to non-PCV13 serotypes 8, 12F, and 9N has increased in England and Wales after the introduction of PCV [4]. However, to the best of our knowledge, there is no report to date of IPD caused by the non-vaccine serotype 28F. The invasion of the pneumococcal serotype 28F into the subcutaneous tissue presented an intriguing question. IPD is usually induced by three steps: transmission, colonization, and invasion [2, 11, 12]. Pneumococci are transmitted to a host from the nasal secretion of the carriers. The transmitted pneumococci colonize the mucosa of the upper respiratory tract of the host and invade sterile sites, such as the bloodstream and cerebrospinal fluid. However, the nasopharyngeal and blood sample cultures of this patient were negative. Furthermore, the patient was afebrile prior to hospitalization.

Since the patient had received a second PCV-13 vaccination on her left thigh one month before hospitalization, vaccination was suspected as the cause of the subcutaneous abscess. The common vaccination-related adverse events include local reactions, such as pain, swelling, erythema, and induration. As vaccines contain several components, such as antigens, adjuvants, conjugates, antimicrobials, stabilizers, and preservatives, acute or delayed-type hypersensitivity reactions against these components can occur [13]. Most of the subcutaneous abscess owing to vaccination are sterile abscesses caused by the delayed-type hypersensitivity reaction to aluminum, an adjuvant in vaccines [14, 15]. A bacterial subcutaneous abscess following vaccination is quite rare [16]. We speculate that bacterial subcutaneous abscess following vaccination had occurred as a result of pathogens that were carried from the skin by the needle or seeded into an injected area [17].

A subcutaneous abscess is a common pediatric skin and soft tissue infection, whereas a subcutaneous abscess caused by *S. pneumoniae* is quite rare since the introduction of PCV [18–21]. More than half of the subcutaneous abscesses are caused by community-acquired methicillin-resistant *Staphylococcus aureus* (CA-MRSA) [18, 19, 22]. Other causative microorganisms include methicillin-sensitive *S. aureus*, coagulase-negative *Staphylococcus*, and *Streptococcus* species, such as group A and group B Streptococcus [18, 19, 23]. Moreover, *Haemophilus influenza type b* (Hib) has also been a causative microorganism, but the incidence of skin and soft tissue infections due to Hib has dramatically decreased after the introduction of the conjugate Hib vaccine [24]. Underlying conditions, such as primary immune deficiencies, HIV, connective tissue disease, and diabetes mellitus are predisposing factors of pneumococcal subcutaneous abscesses [25]. Because the patient did not have any of these underlying conditions, the abscess may be attributed to the high virulence of the serotype 28F.

We could not distinguish between subcutaneous abscess and cellulitis by clinical examination alone in this patient, but ultrasonographic examination was useful for differentiating between the two diseases. As the symptoms of subcutaneous abscess and cellulitis overlap clinically, the diagnosis of subcutaneous abscess based on clinical examination alone is sometimes difficult. A subcutaneous abscess requires incision and drainage, whereas cellulitis is treated with antibiotics alone [26, 27]. Therefore, accurate differentiation can reduce unnecessary drainage procedures, sedation, and costs [28, 29]. In a systematic review that evaluated the accuracy of ultrasonography for the diagnosis of subcutaneous abscess in pediatric patients, the sensitivity and specificity were 94.9% and 83.1%, respectively [30]. Therefore, ultrasonography is useful in differentiating between subcutaneous abscess and cellulitis.

As the patient was a 4-month-old infant and had a large subcutaneous abscess, she was hospitalized for parenteral therapy. In skin and soft tissue infections, hospitalization for parenteral therapy is recommended for patients with a large abscess, fever, other signs of systemic infection, or high-risk characteristics, such as age younger than 6 months, diabetes, or immunodeficiency [31]. The susceptibility to penicillin differs for various *S. pneumoniae* serotypes [7–10]. The serotype 28F was susceptible to penicillin, cephalosporins, carbapenems, quinolones, and macrolides.

In conclusion, subcutaneous abscess is a common pediatric skin and soft tissue infection, whereas pneumococcal subcutaneous abscesses are quite rare. As the pneumococcal serotype 28F caused a subcutaneous abscess, this serotype possibly has a high virulence. The incidence of IPD caused by non-vaccine serotypes, such as 28F, is expected to increase in the future. The consolidation of international data on pneumococcal serotypes is important for the development of novel PCV that can be designed to prevent serotype replacement.

## Data Availability

Not applicable.

## Abbreviations

IPD: Invasive pneumococcal disease
PCV: Pneumococcal conjugate vaccines
PCV-7: Seven-valent PCV
PCV-13: 13-valent PCV
PAPM/BP: Panipenem/betamipron
CA-MRSA: Community-acquired methicillin resistant *Staphylococcus aureus*
Hib: *Haemophilus influenza type b*
